# Refusal of treatment for acute leukemia in pregnancy: a case report

**DOI:** 10.1186/1752-1947-7-148

**Published:** 2013-05-31

**Authors:** Syheda Latifi Hoxha, Vlora Ademi Ibishi, Ahmet Brovina, Mynevere Hoxha, Shefqet Lulaj

**Affiliations:** 1 Obstetrics and Gynecology Clinic, University Clinical Center of Kosova, Prishtina, 10000, Kosova; 2 Hematology Clinic, University Clinical Center of Kosova, Prishtina, 10000, Kosova

**Keywords:** Chemotherapy, Coagulopathy, Leukemia, Pregnancy

## Abstract

**Introduction:**

Acute leukemia is rare in pregnancy. The importance of promptly diagnosing and treating this disease in pregnancy stems from its life-threatening potential, both to the mother and fetus.

**Case presentation:**

We report a case of relapse of acute myeloid leukemia at 23 weeks of pregnancy in a 24-year-old Albanian woman. Our patient categorically refused chemotherapy treatment, and in her 35th week of gestation, severe hemorrhagic diathesis rapidly developed. The manifestation and course of this life-threatening complication posed therapeutic challenges for the attending medical team.

**Conclusion:**

Based both on our experience and the results of other gynecological studies, there exists a strong indication that the earlier a patient’s chemotherapy treatment begins, the better the maternal outcome. We support chemotherapy for patients who are pregnant presenting with such illness. The present case report testifies that refusal of chemotherapy by such patients is a high-risk decision.

## Introduction

One in 1000 pregnancies coexist with malignant disease [1,2]. The incidence of leukemia in pregnancy is one in 75,000 to 100,000 pregnancies [3]. The majority of cases of leukemia diagnosed during pregnancy are acute [4-6]. Acute leukemia normally requires prompt treatment despite significant risks of pregnancy loss and birth defects, especially if chemotherapy is given during the first trimester of pregnancy. Evidence suggests that treatment postponement until the post-partum period is associated with increased maternal mortality [7].

The association of malignancy with pregnancy always creates a serious dilemma for both the pregnant woman and the medical team. Although rare, evidence from case reports of patients who are pregnant who have acute leukemia, including cases of relapse, provides very helpful treatment information in such medical dilemmas.

## Case presentation

At 23 weeks of gestation, a pregnant 24-year-old Albanian woman, gravida 3, para 2, was admitted to our hematology clinic with chest pain, nosebleeds, gingival bleeding, general weakness, and fatigue. One year prior to admission, she had been diagnosed as having acute myeloid leukemia and was treated with doxorubicin and cytarabine according to our chemotherapy protocol.

Six months after chemotherapy, while in remission, she became pregnant. Her pregnancy progressed with no complications until the 23rd week of gestation. Her general health became impaired, and laboratory test results confirmed a relapse of acute myeloid leukemia. Her obstetric examination findings and fetal sonography results were normal and appropriately correlated with the gestational age. Blood count results revealed that her maternal hemoglobin (Hgb) level was 8.5g/dL, platelet (Plt) count was 65×10^9^plt/L, red blood cell (RBC) count was 2.8×10^9^ cells/L, white blood cell (WBC) count was 45×10^9^ cells/L with 50 percent blast cells, hematocrit (Hct) was 29 percent, and sedimentation rate was 35mm. Her urea and creatinine levels were within the normal ranges.

The attending medical team included a hematologist, an oncologist, and an obstetrician. After analyzing our patient’s history, medical examination results, and laboratory data, the medical team proposed that our patient begin chemotherapy treatment without delay. In spite of the medical team’s advice and consultations with our patient, she categorically refused any kind of chemotherapy treatment. Our patient’s decision was therapeutically challenging for the medical team.

Our patient was frequently followed up with clinical assessments and laboratory tests. At 32 weeks of gestation, urine culture results showed an *Escherichia coli* infection, which was treated with ceftriaxone. A blood count performed at this time revealed a RBC count of 2.5×10^9^ cells/L, WBC count of 30.8×10^9^ cells/L, Plt count of 41×10^9^plt/L, Hgb level of 6.6g/dL, and Hct of 22.2 percent.

Supportive therapy including fresh whole blood and blood product transfusions was administered to stabilize our patient. The fetal condition was closely monitored using ultrasound and later using cardiotocography. At 34 weeks of gestation, a course of betamethasone was administered. The ultrasound measurements showed slight intra-uterine growth restriction, but no fetal distress.

At 35 weeks of gestation, our patient’s general health declined and there was evidence of fetal distress. Laboratory test results at this time showed the following alterations: RBC, 3.1×10^12^ cells/L; WBC, 38.6×10^9^ cells/L; Hgb, 10g/dL; Hct, 28 percent; Plt, 10×10^9^plt/L; lymphocytes, 12.8 percent; monocytes, 10.4 percent; and granulocytes, 76.8 percent. Coagulation analysis revealed a prothrombin time of 16.8 seconds (reference range, 9.8 to 13.4 seconds) and an activated partial thromboplastin time of 24.0 seconds (reference range, 27.9 to 41.6 seconds).

**Figure 1:**
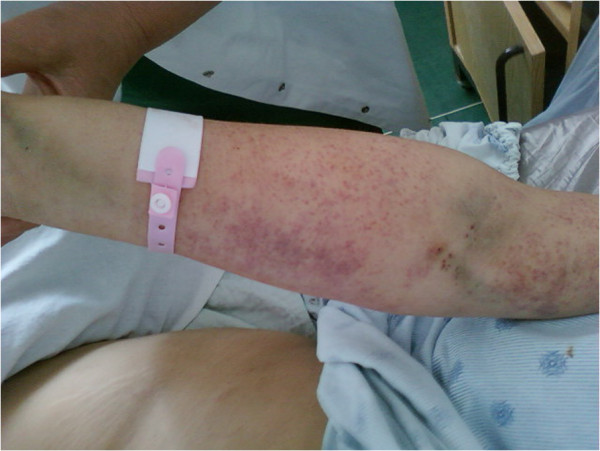
Cutaneous bleeding manifestations.

**Figure 2:**
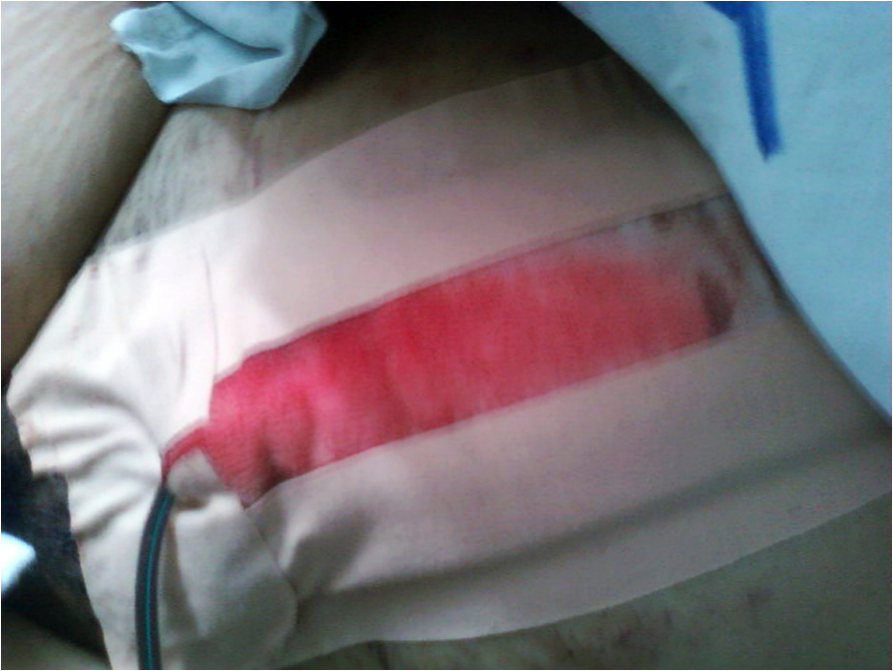
**Operative wound bleeding.** Two hours after the Cesarean section, our patient developed the following hemorrhagic manifestations: gingival bleeding, generalized purpura and petechiae, and operative wound bleeding. Laboratory test results showed a platelet count of 8×10^9^plt/L.

An emergency Caesarean section was performed, and a healthy baby was delivered (weight, 1900g; Apgar score, 5/6). During the operation, 1U of fresh whole blood and 3U of fresh plasma were transfused because of intra-operative bleeding. Then, two hours after the Caesarean section, our patient developed generalized purpura (Figure 1) and petechiae, gingival bleeding, and operative wound bleeding (Figure 2). Approximately 300mL of blood was drained from the Douglas pouch. Laboratory test results showed a Plt count of 8×10^9^plt/L. After consulting with our hematologist, we administered another 2U of blood, 8U of fresh plasma, and 15U of platelets. Five days after the Cesarean section, our patient was transferred to our Hematology Clinic, where she agreed to undergo chemotherapy.

Six days after delivery, she began chemotherapy comprising an anti-metabolite and anthracycline, namely cytarabine (200mg/m^2^ for seven days) and doxorubicin (60mg/m^2^ for three days), respectively. Close neonatal follow-up revealed normal development. To date, our patient remains in complete remission, and the development of her baby, now three years of age, has progressed with no complications.

## Discussion

The actual incidence of leukemia in pregnancy is not well known. It is estimated to range from one in 75,000 to 100,000 pregnant women [8]. Most cases of leukemia associated with pregnancy are acute, while less than 10 percent of all cases are chronic. Among cases of acute leukemia, myeloid leukemia occurs twice as often as acute lymphoblastic leukemia.

Three unique subgroups of acute myeloid leukemia are recognized by the World Health Organization classification (Table 1): (1) acute myeloid leukemia with recurrent genetic abnormalities; (2) acute myeloid leukemia with multi-lineage dysplasia; and (3) therapy-related acute myeloid leukemia and myelodysplastic syndrome. Cases that do not satisfy the criteria for any of these subgroups or for which no genetic data can be obtained are classified into a fourth subgroup: acute myeloid leukemia, not otherwise categorized [9].

**Table 1:** World Health Organization classification of acute myeloid leukemia

**Subgroup**	**Type**
Acute myeloid leukemia with recurrent genetic abnormalities	Acute myeloid leukemia with t(8;21)(q22;q22), (AML1/ETO)cpn
	Acute myeloid leukemia with abnormal bone marrow eosinophils and inv(16)(p13q22) or t(16;16)(p13;q22), (CBFβ/MYH11)
	Acute promyelocytic leukemia with t(15;17)(q22;q12), (PML/RARα), and variants
	Acute myeloid leukemia with 11q23 (MLL) abnormalities
Acute myeloid leukemia with multi-lineage dysplasia	Following myelodysplastic syndrome or myelodysplastic syndrome/myeloproliferative disease
	Without antecedent myelodysplastic syndrome or myelodysplastic syndrome/myeloproliferative disease, but with dysplasia in at least 50 percent of cells in two or more myeloid lineages
Therapy-related acute myeloid leukemia and myelodysplastic syndrome	Alkylating agent/radiation-related type
	Topoisomerase II inhibitor-related type (some may be lymphoid)
	Others
Acute myeloid leukemia not otherwise categorized	Acute myeloid leukemia, minimally differentiated
	Acute myeloid leukemia without maturation
	Acute myeloid leukemia with maturation
	Acute myelomonocytic leukemia
	Acute monoblastic/acute monocytic leukemia
	Acute erythroid leukemia (erythroid/myeloid and pure erythroleukemia)
	Acute megakaryoblastic leukemia
	Acute basophilic leukemia
	Acute panmyelosis with myelofibrosis
	Myeloid sarcoma

Acute myeloid leukemia may be associated with life-threatening hemorrhagic diathesis, which is attributed to disseminated intra-vascular coagulation. Disseminated intra-vascular coagulation may complicate the course of leukemia in as many as 50 percent of patients [10]. The most common leukemia associated with disseminated intra-vascular coagulation is acute promyelocytic leukemia followed by acute myelomonocytic leukemia, acute myeloblastic leukemia, and acute lymphoblastic leukemia [11].

Acute leukemia is an extremely aggressive disease and is fatal unless promptly treated [12]. During pregnancy, acute myeloid leukemia poses an immediate threat to the lives of both the mother and fetus, making prompt diagnosis and treatment extremely important [13]. Acute leukemia requires immediate treatment, regardless of gestational age. Patients with leukemia should undergo aggressive chemotherapy until remission is achieved, regardless of pregnancy. In cases of relapse, the chemotherapy should be started without delay. Chemotherapy during the first trimester is associated with an increased risk of congenital malformations; thus, abortion (termination of the pregnancy) should be considered. Standard anti-leukemia treatment can be safely administered during the second and third trimesters of pregnancy [14].

Evidence indicates that maternal and fetal outcomes improve substantially with medical treatment. Therefore, advocating such chemotherapy treatments would be both physically beneficial for pregnant women as well as helpful for mothers during their decision-making process.

## Conclusions

Our patient’s case is an example of fertility preservation following anti-leukemia chemotherapy. Our patient became pregnant during her remission phase, six months after completion of chemotherapy. Our patient’s case also suggests that pregnancy might be associated with an increased risk of acute leukemia relapse. The maternal decision to categorically refuse chemotherapy despite the associated risks also underlines the therapeutic challenge faced by the attending medical team in such cases. In light of other reports that have documented similar successfully treated cases, along with our experience of treatment refusal followed by life-threatening hemorrhagic diathesis, we promote chemotherapy for patients who are pregnant who are diagnosed as having acute myeloid leukemia.

## Consent

Written informed consent was obtained from the patient for publication of this manuscript and any accompanying images. A copy of the written consent is available for review by the Editor-in-Chief of this journal.

## Competing interests

The authors declare that they have no competing interests.

## Authors’ contributions

SLH, VAI, MH, AB, and SL participated in the development of this manuscript. SLH, VAI, and SL provided obstetric care to our patient. SLH was the main obstetrician during the Cesarean section. AB analyzed and interpreted the data from our patient regarding hematological disease. MH was primarily involved in the care of our patient’s child. SLH, VAI, AB, and SL were involved in the writing of the manuscript. SLH, VAI, AB, and MH were involved in editing of the manuscript. All authors read and approved the final manuscript.
